# Gelatin Enhances the Wet Mechanical Properties of Poly(D,L-Lactic Acid) Membranes

**DOI:** 10.3390/ijms25095022

**Published:** 2024-05-04

**Authors:** Deuk Yong Lee

**Affiliations:** 1 Department of Biomedical Engineering, Daelim University, Anyang 13916, Republic of Korea; duke1208@gmail.com; 2 Department of R&D Center, Hass Co., Ltd., Gwangmyeong 14322, Republic of Korea

**Keywords:** poly(D,L-lactic acid) (PDLLA), gelatin, electrospinning, wet mechanical properties, absorbable periodontal bone regeneration, biocompatibility

## Abstract

Biodegradable (BP) poly(D,L-lactic acid) (PDLLA) membranes are widely used in tissue engineering. Here, we investigate the effects of varying concentrations of PDLLA/gelatin membranes electrospun in 1,1,1,3,3,3-hexafluoro-2-propanol (HFIP; C_3_H_2_F_6_O) solvent on their mechanical and physical properties as well as their biocompatibility. Regardless of the environmental conditions, increasing the gelatin content resulted in elevated stress and reduced strain at membrane failure. There was a remarkable difference in strain-to-failure between dry and wet PDLLA/gelatin membranes, with wet strains consistently higher than those of the dry membranes because of the hydrophilic nature of gelatin. A similar wet strain (ε_w_ = 2.7–3.0) was observed in PDLLA/gelatin membranes with a gelatin content between 10 and 40%. Both dry and wet stresses increased with increasing gelatin content. The dry stress on PDLLA/gelatin membranes (σ_d_ = 6.7–9.7 MPa) consistently exceeded the wet stress (σ_w_ = 4.5–8.6 MPa). The water uptake capacity (WUC) improved, increasing from 57% to 624% with the addition of 40% gelatin to PDLLA. PDLLA/gelatin hybrid membranes containing 10 to 20 wt% gelatin exhibited favorable wet mechanical properties (σ_w_ = 5.4–6.3 MPa; ε_w_ = 2.9–3.0); WUC (337–571%), degradability (11.4–20.2%), and excellent biocompatibility.

## 1. Introduction

Poly(D,L-lactic acid) (PDLLA), a biodegradable polymer (BP) with high mechanical strength, is widely used in tissue engineering applications such as scaffolds and drug delivery systems [1,2,3,4,5,6,7]. In conventional drug delivery systems, owing to the degradation pattern, drug release is sustained over time; therefore, when infections occur, the local level of antibiotics may fail to reach the effective therapeutic dose [4,5]. An ideal scaffold should degrade at a rate similar to that at which the cells generate the extracellular matrix (ECM). PDLLA-modified scaffolds allow for a controlled drug delivery rate over a relatively long period [4,5,6]. PDLLA membranes, pins, and screws used in dental guided bone regeneration (GBR) are composed of a 50/50 mixture of the D and L isomers of poly lactic acid (an equimolar racemic mixture) to extend absorption time and improve mechanical properties [1,2,7]. The hydrophobicity of PDLLA BP can be tuned with surface modification via sputtering [8], hydrothermal treatment [9], or alloying with natural polymers [7,10,11,12,13,14,15]. In addition to hydrophilic tuning, alloying PDLLA membranes with natural polymers allows for the regulation of PDLLA membrane mechanical properties and biological functions [7,10,11,12,13]. In our previous study, we demonstrated that a limited amount (0–4 wt%) of gelatin could be alloyed into PDLLA using eco-friendly solvents consisting of acetic acid, ethyl acetate, and distilled water [7]. Compared to commercial membranes, the tensile stress (3.9 ± 0.7 MPa), strain at break (0.37 ± 0.01), and water uptake capacity (WUC, 273 ± 33%) of the PDLLA/3 wt% gelatin membrane were insufficient [7,10]. Owing to the slow solidification of the aliphatic polymer chains in a green solvent, PDLLA/gelatin hybrid membranes exhibit relatively poor properties, including poor strain-to-failure and WUC [7]. To improve the performance of the membrane, a rapid chemical reaction was performed using a fluorinated alcohol solvent (1,1,1,3,3,3-hexafluoro-2-propanol, HFIP, or C_3_H_2_F_6_O). Fluorine attracts the negative charge of the conjugate base through a six-sigma bond and delocalizes the electron density, rendering it a strong acid and improving its mechanical properties [10]. The synthetic graft barrier used for GBR induction is prepared via electrospinning in the HFIP solvent [1,10]. Overall, the hydrophobicity of PDLLA was modified by alloying it with gelatin [7]; however, the optimal PDLLA/gelatin content remains unknown. Therefore, in this study, we aimed to determine the effect of electrospinning PDLLA/gelatin membranes (varying concentrations) in HFIP solvent on the mechanical and physical properties and biocompatibility of PDLLA/gelatin membranes.

## 2. Results and Discussion

**Figure 1:**
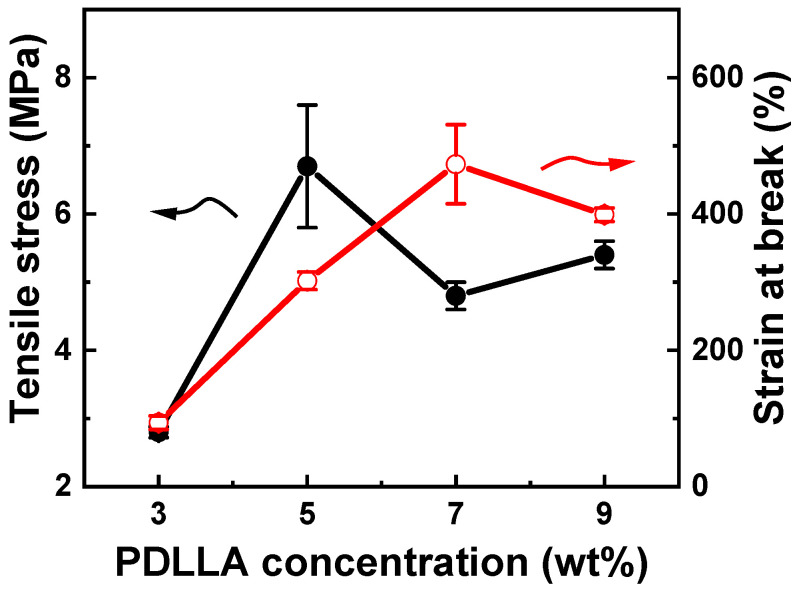
Mechanical properties of poly(D,L-lactic acid) (PDLLA) membranes as a function of PDLLA concentration.

The tensile strength and strain-to-failure of the PDLLA membranes in the concentration range of 3–9 wt% are shown in Figure 1. Owing to their lower viscosity (73 cP), bead-on-string-structured nanofibers were only found in 3 wt% PDLLA [16]. As the PDLLA concentration increased from 3 wt% to 5 wt%, the tensile strength increased dramatically from 2.8 ± 0.1 MPa to 6.7 ± 0.9 MPa. However, as the PDLLA concentration increased from 5 wt% to 9 wt%, the tensile strength decreased from 6.7 ± 0.9 MPa to 5.4 ± 0.2 MPa. Owing to its suitable strength (6.7 ± 0.9 MPa) and strain at failure (302 ± 13%), a 5 wt% PDLLA membrane was selected. However, the WUC of the 5 wt% PDLLA membrane was 57%, which was insufficient for a GBR barrier membrane [10].

Thus, owing to its excellent hydrophilicity, biocompatibility, low antigenicity, unique arginine–glycine–aspartate motif, and cell affinity, gelatin was used in PDLLA to improve the WUC of the PDLLA membrane [17,18,19,20,21]. Gelatin, a polypeptide (triple-helix conformation) derived from collagen hydrolysis, has been used as a biological polymer because of its low water solubility and immunogenicity [10,11,12,17,18,19,20,21]. Interchain hydrogen bonds form triple helices that stabilize and entangle the collagen structures. After the hydrogen and covalent bonds break, the triple helix becomes gelatinous along the coil structure [17,18]. A blend of hydrophilic single and double unfolded chains offers excellent physical properties such as gelatinous force, high affinity, low viscosity, dispersion stability, and water-holding capacity [17,18,19,20,21]. PDLLA/gelatin hybrid membranes exhibit tunable physical properties and biological functions [7,10,11,20,21,22,23]. Thus, the properties of the electrospun PDLLA/gelatin membranes with a polymer concentration of 5 wt% were investigated as a function of the gelatin concentration from 0 to 40 wt%.

**Figure 2:**
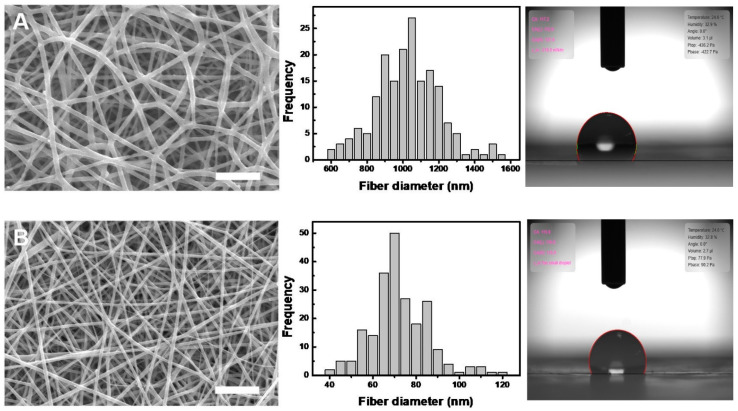

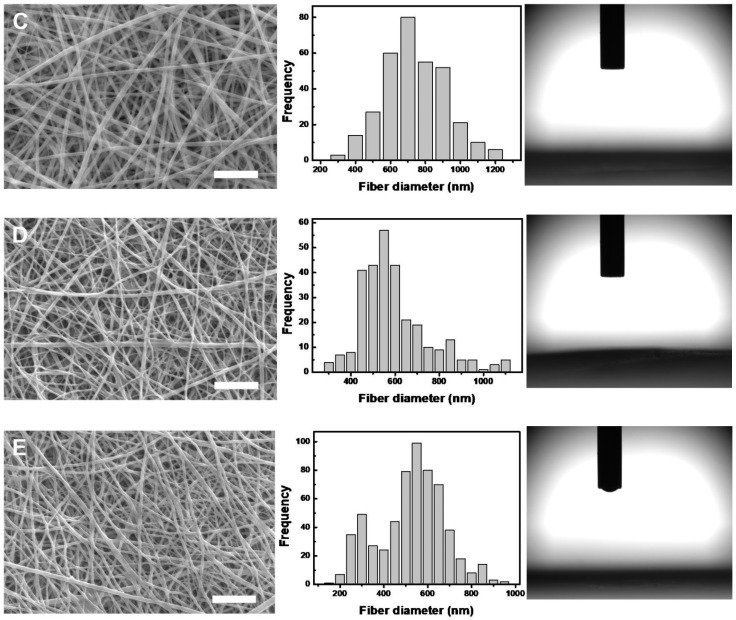
Scanning electron microscopy images, histograms, and water absorption capacity of the 5 wt% poly(D,L-lactic acid)/gelatin membranes as a function of gelatin concentration: (**A**) 0; (**B**) 10; (**C**) 20; (**D**) 30; and (**E**) 40 wt%. Scale bar = 10 μm.

**Figure 3:**
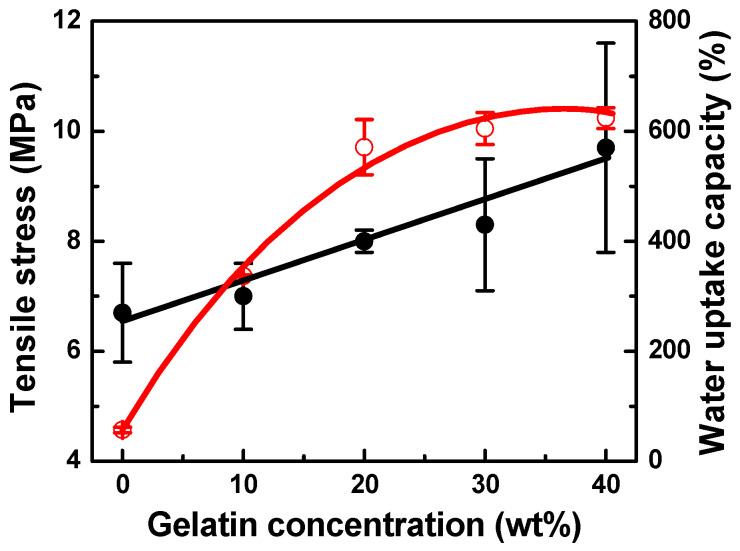
Changes in tensile stress and water uptake capacity of poly(D,L-lactic acid)/gelatin membranes as a function of gelatin concentration. Note that solid and open circles represent tensile stress and WUC.

SEM images of the electrospun 5 wt% PDLLA/gelatin membranes with gelatin contents ranging from 0 to 40 wt% are shown in Figure 2. Gelatin molecules with good dielectric constants are easily charged during electrospinning [10,21,22,23,24,25]. Therefore, electrospun jets with a higher gelatin content have a higher surface charge and are likely to exert stronger stretching forces, resulting in thinner fibers [20,21,22,23,24,25]. As the solution conductivity or charge density increases, the fibers become thinner [20,21,22,23,24,25]. As the gelatin concentration increased from 0 to 10 wt%, the fiber diameter decreased from 746 to 533 nm and then reached and maintained a steady state, even with gelatin doping. However, as the gelatin content increased from 0 to 40 wt%, the PDLLA/gelatin membrane failure strain decreased rapidly from 3.02 to 0.35. Nanofibrous PDLLA/gelatin membranes at certain ratios exhibited a better WUC; as the gelatin content increased from 0 to 10 wt%, the water contact angle (WCA) decreased from 132 ± 3° to 120 ± 5°, whereas the WUC increased from 57% to 337%. When the gelatin content exceeded 20%, the WCA was 0. Yan et al. reported that the stress of a pure nanofibrous gelatin membrane was 1.9 MPa [21]. The stress and failure strain of the PLLA/gelatin membrane increased to 3.48 MPa and 0.57, respectively, as the ratio of PLLA to gelatin increased to 7. Additionally, with an increase in gelatin content from 0 to 40 wt%, the tensile stress of PDLLA/gelatin increased from 6.7 ± 0.9 MPa to 9.7 ± 1.9 MPa (Figure 3). In addition, the addition of gelatin (20%) increased the WUC of the PDLLA/gelatin blend from 57% to 571%. The strength, failure strain, and WUC of a commercially available absorbable synthetic graft membrane composed of PLLA and collagen were reported to be 3.7 ± 0.5 MPa, 0.55, and 455%, respectively [10]. As the gelatin concentration increased from 0% to 10%, WUC increased significantly from 57% to 337% (Figure 3). The tensile stress, strain at break, and WUC of 90/10 and 80/20 PDLLA/gelatin membranes at 5 wt% concentration were 7.0 ± 0.6 MPa, 2.1 ± 0.2, 337 ± 2%, and 8.0 ± 0.2 MPa, 0.8 ± 0.4, 571 ± 50%, respectively. Owing to their excellent mechanical properties and WUC, both membranes were suitable for GBR. 

**Figure 4:**
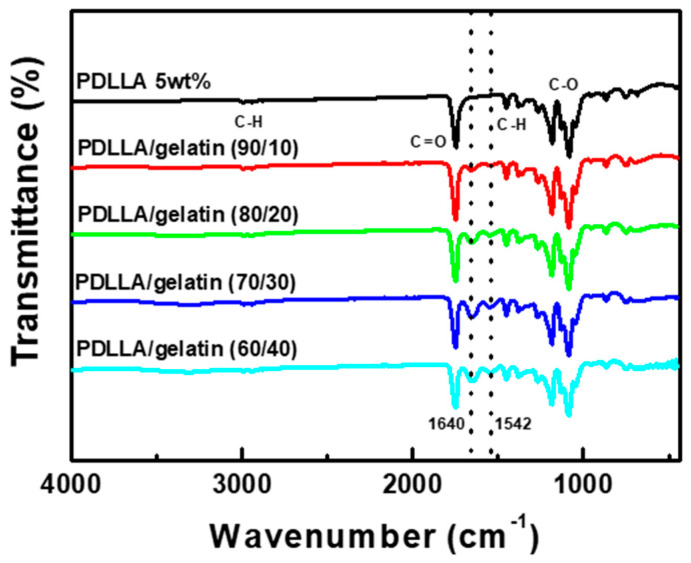
Fourier transform infrared spectra of various poly(D,L-lactic acid) (PDLLA)/gelatin membranes.

The Fourier transform infrared spectra of the PDLLA/gelatin membranes with different gelatin concentrations are shown in Figure 4. PDLLA is a racemic mixture of PDLA and PLLA enantiomers with a chiral structure containing four ligands [1,7,25]. The characteristic C=O peak (1754 cm^−1^) of PDLLA was clearly observed for the carboxylic acid, carbonyl moieties, and methyl groups (–CH_3_, 2995 cm^−1^) [7,25]. The bands at 1080 and 1185 cm^−1^ and 1453 and 1382 cm^−1^ correspond to C–O stretching and C-H bending vibrations, respectively [7,25]. When gelatin was blended with PDLLA, amide peaks (1640 cm^−1^ and 1542 cm^−1^) representing gelatin were identified [7,17,18]. However, no new absorption peaks appeared for the PDLLA/gelatin membranes, indicating that no chemical reactions occurred between the PDLLA and gelatin. PDLLA and gelatin molecular chains interact only via van der Waals forces [7,10]. Similar results indicating non-covalent bonding were observed for PLLA/gelatin BP membranes [10,20,21].

**Figure 5:**
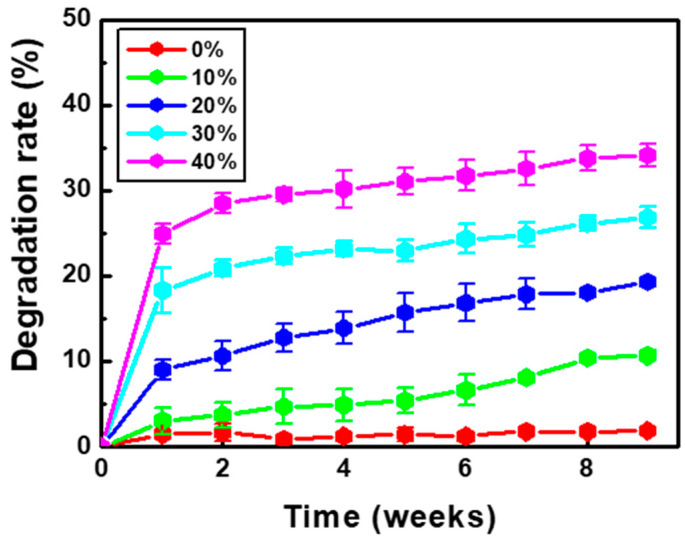
Enzymatic degradation rate of poly(D,L-lactic acid)/gelatin membranes as a function of gelatin concentration.

**Figure 6:**
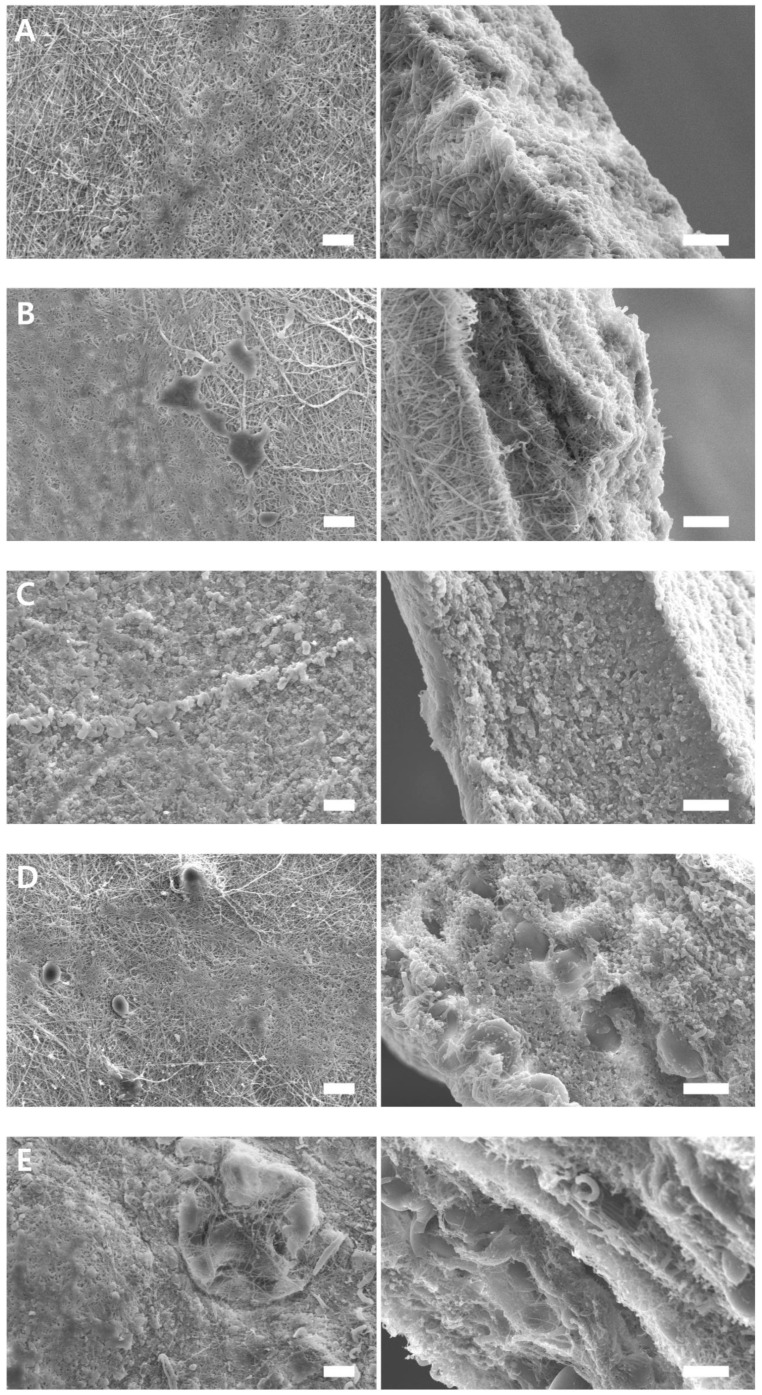
Surfaces and cross-sectional scanning electron microscopy images of poly(D,L-lactic acid)/gelatin membranes at gelatin concentrations of (**A**) 0%, (**B**) 10%, (**C**) 20%, (**D**) 30%, and (**E**) 40%, 8 weeks after degradation. Scale bar = 20 μm.

There are many opinions about the appropriate time for barrier resorption, but 3–6 weeks is an important period for the surrounding healthy periodontal ligament cells to settle and new tissue to mature; therefore, destruction of the barrier membrane should not occur within this period [26,27,28]. No inflammatory cells or giant cells were observed. BP PDLLA generally decomposes in two stages when exposed to the human body [1]. Initially, water molecules hydrolyze the long polymer chains into shorter fragments via ester bond cleavage. These fragments are then metabolized to produce carbon dioxide and water, which are eventually excreted through respiration [1]. The degradation rates of the PDLLA/gelatin membranes are shown in Figure 5. Over time, PDLLA degradation (1.8%) was not significant. However, owing to increased hydrolysis, the degradation of PDLLA with a high gelatin content increased dramatically. The degradation rate increased rapidly in week 1 but slowed down after week 2. After 8 weeks, as the gelatin content increased from 0 to 40%, the degradation rate increased drastically from 1.8% to 34%. Similar degradation characteristics (38%) were observed for the PLLA/40 wt% gelatin membrane [10]. However, no PDLLA/gelatin membrane fragmentation was observed, probably because of the higher PDLLA content. PDLLA/gelatin membrane surface and cross-sectional SEM images taken 8 weeks post-degradation are shown in Figure 6. Intermittent and sparse aggregation was initially observed. As the gelatin content increased, the water and enzyme aggregated and spread across the membrane surface. Weak bonds are cleaved during the breakdown of hydrolytically unstable polymer chains, producing smaller polymer fragments [5]. Owing to smaller fragments, membrane degradation (34%) was observed on the surface of the PDLLA/gelatin membrane, which contained 40% gelatin. Cross-sectional images of the membranes show that as the gelatin content increased from 0 to 40% the morphology changed from a fibrous, smooth, defect-free structure to a structure that was severely broken and split by defects. Increased enzymatic degradation significantly affected PDLLA/gelatin membrane morphology.

The cytotoxicity of PDLLA/gelatin membranes with gelatin concentrations ranging from 0 to 40% was determined [10,29,30,31,32]. L-929 and MG-63 cell viability on PDLLA/gelatin membranes containing 0, 10, 20, 30, and 40% gelatin were 108, 106, 106, 109 and 103%, and 93.8, 107, 99.0, 119, and 124%, respectively (Figure S1).

**Figure 7:**
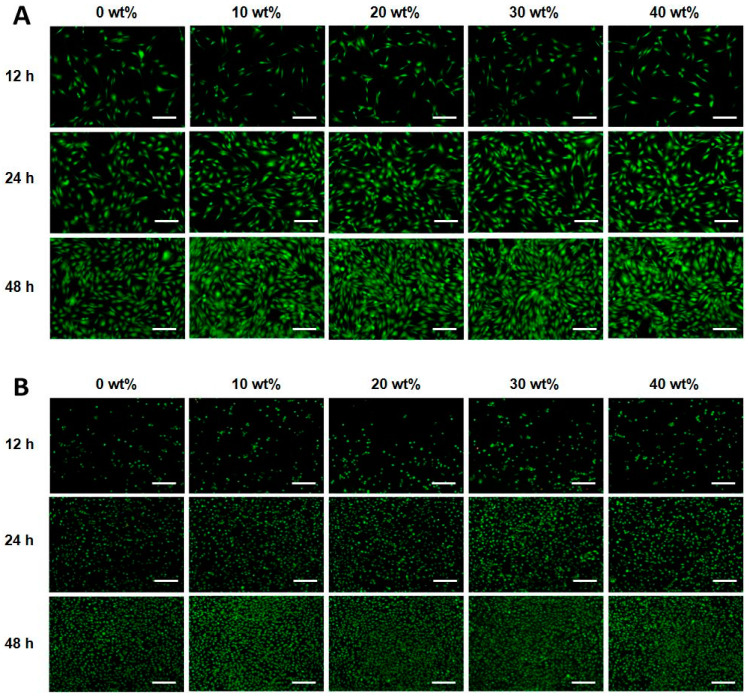
Fluorescence microscopy images of live/dead assays showing (**A**) MC3T3-E1 and (**B**) L-929 cells incorporated poly(D,L-lactic acid)/gelatin membranes containing varying amounts of gelatin after 12, 24, and 48 h. Scale bar = 200 μm and amplification = 10×.

Cell viability was further examined at different incubation times (12, 24, and 48 h). Viable MC3T3-E1 and L-929 cells (stained green) and dead cells (stained red) were visualized using fluorescence microscopy. At 24 h, MC3T3-E1 and L-929 cells were stained green throughout the membrane, indicating significant cell proliferation. The cells proliferated continuously over time, indicating that the PDLLA/gelatin membranes are not cytotoxic, rendering the membranes suitable for bone regeneration applications [10,29,30,32]. PLLA/gelatin membranes containing gelatin (10–20%) are suitable as barrier membranes for absorbable GBR because of their commercially available mechanical properties and biocompatibility [32,33,34]. The synergistic combination of structural integrity and modified hydrophilicity may be effective for periodontal barrier biomaterials that require rapid healing regeneration [7,10,32,33,34,35,36]. Regardless of the gelatin content, all PDLLA/gelatin membranes showed cell viability of more than 90%, indicating no cytotoxicity [7,10,29,32,33,34,35,36]. MC3T3-E1 and L-929 cellular proliferations on PDLLA/gelatin membranes are depicted in Figure 7. Owing to its customized mechanical properties and biocompatibility, the PDLLA/gelatin membrane containing 10–20% gelatin serves as an attractive barrier membrane for resorbable periodontal regeneration [33,34,35].

PDLLA/gelatin BP membranes can be used for GBR in dentistry to correct bone defects in areas treated with implants. If a tooth is lost, an implant is placed on the alveolar ridge filled with bone graft material, and a barrier membrane is placed in a shielding form to protect the bone graft material from rapidly growing gingiva. As a periodontal regeneration barrier is present within the oral cavity, wettability is important. Conventionally, periodontal barrier membranes, sutures, pins, and screws are soaked in the appropriate saline solution before use [1,10,27,28,33,34]. However, stiffness is usually reduced in wet conditions, resulting in the loss of space between the barrier and tooth, which can be detrimental to clinical outcomes [10]. The finger-stretched optical photographs of various PDLLA/gelatin membranes under dry and wet conditions are shown in Figure S2. The extension properties of the PDLLA/gelatin membranes in the dry state decreased dramatically with increasing gelatin concentrations from 0 to 40%, which is in good agreement with the strain results upon failure. However, better extension was observed when the membrane was wet, regardless of the gelatin content. The presence of gelatin has been attributed to its greater extension, as it can retain large amounts of water within the network without dissolving or disrupting its structural integrity [10,17,18]. As shown in Figure S3, as the gelatin content increased to 20 wt%, the optical properties of the wet membrane changed from opaque to translucent. However, owing to the absence of water molecules, no noticeable differences were observed in the optical properties of the dried membranes, regardless of the gelatin concentration.

**Figure 8:**
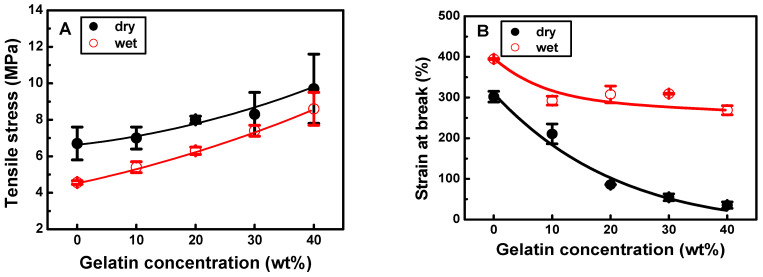
(**A**) Tensile stress and (**B**) strain at break of various poly(D,L-lactic acid)/gelatin membranes in dry and wet conditions.

The dry and wet mechanical properties of the PDLLA/gelatin membranes are shown in Figure 8. Regardless of the environment, the stress and strain at membrane break increased and decreased, respectively, with increasing gelatin content. There was a significant difference in the strain-to-failure between the dry and wet PDLLA membranes. Although both wet and dry strains decreased with increasing gelatin content, the wet strains (ε =2.7–3.9) on the PDLLA/gelatin membranes were always higher than those (ε = 0.35–3.0) on the dry membranes. This may be due to the hydrophilic nature of gelatin because it can hold large amounts of water within the network without collapsing the structure [17,18]. Similar wet strains (ε = 2.7–3.0) of PDLLA/gelatin membranes with gelatin content between 10% and 40% may occur, probably due to insufficient soaking time [10]. However, the failure strain of the PDLLA/gelatin membrane is much higher than that of commercial membranes (ε = 0.12–0.76) [32]. In contrast, dry and wet stresses increased with increasing gelatin content. During hydrolysis, water molecules are preferentially incorporated into amorphous PDLLA, and chain scission reactions are favored within the PDLLA [7,18]. The initiation of ester bond cleavage via hydrolysis is responsible for stress reduction [7,10,17]. Therefore, the wet stress on the PDLLA/gelatin membrane was always lower than the dry stress. If the WUC is good, saliva and blood are absorbed into the intestinal wall during the procedure. It is quickly absorbed and enables the expression of cytokines to produce osteoclasts/osteoblasts, helping to create new bone and restore gingival tissue [20,32,36]. In particular, the sutured area of periodontal-tissue-regeneration-inducing material following gum restoration can be restored in a short time and protected from infection via foreign substances present in saliva [36]. The WUC is also an important factor when drug loading is required. The tensile stress, strain at failure, and WUC of commercial collagen products are 20.5 ± 2.1 MPa, 0.12, and 126%, respectively, indicating low ductility and poor hydration [32]. Pericardium-derived or peritoneal-derived xenograft barriers were 3.4 ± 0.2 MPa, 0.36, and 590% and 15.9 ± 1.9 MPa, 0.76, and 265%, respectively [32]. However, they are expensive. Synthetic commercial graft barriers were reported to be 3.4 ± 0.2 MPa, 0.55, and 455% [32]. PDLLA/gelatin hybrid membranes can be used as GBR inducers regardless of the gelatin content due to their suitable wet mechanical properties (σ = 5.4–8.6 MPa, ε = 2.7–3.0), WUC (337–624%), variable degradability (1.98–34%), and excellent biocompatibilities [1,33,34,35,36]. The absorbable periodontal regeneration barrier manufactured by alloying BP with natural polymers, such as gelatin, starch, and cellulose, is expected to be sufficiently competitive in the market if combined with bone graft materials [1,10,32,33,34,35,36].

**Figure 9:**
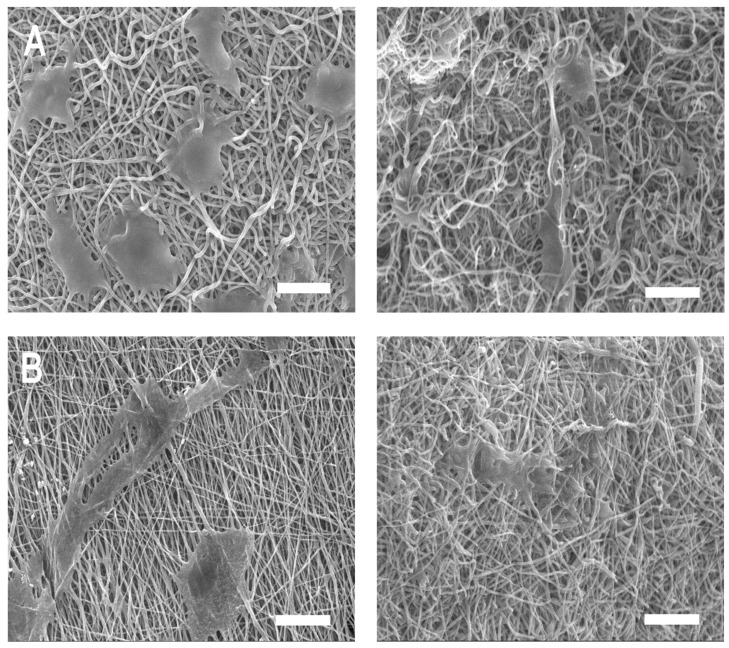

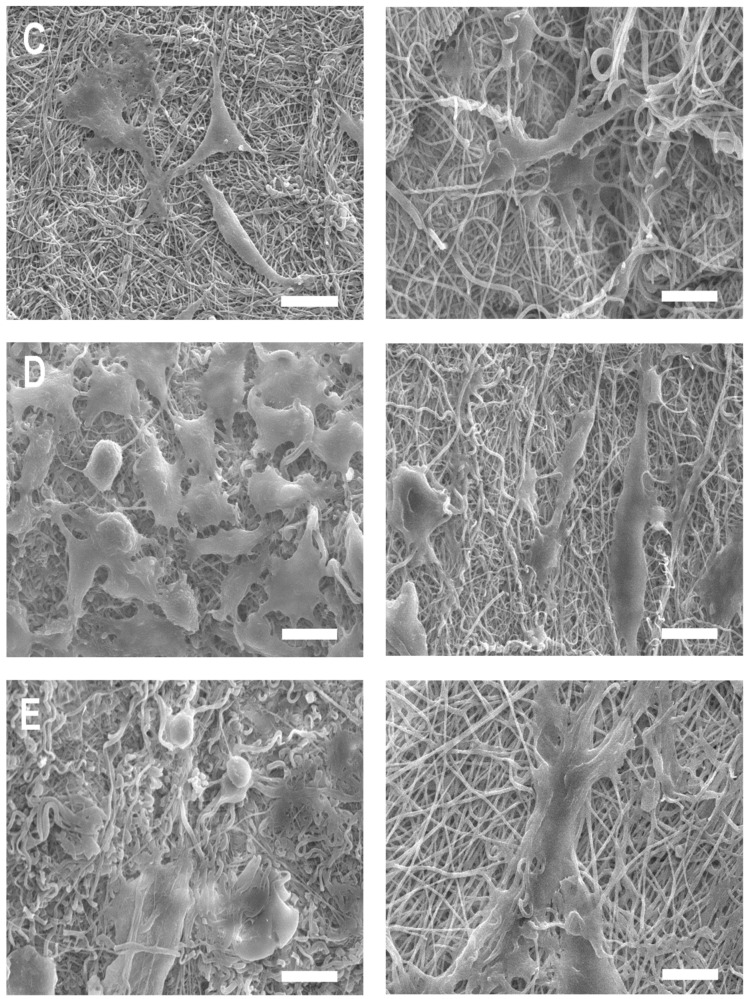
Scanning electron microscopy images of L-929 and MG-63 cell attachment (scale bar: 20 μm) of (**A**) poly(D,L-lactic acid) (PDLLA), (**B**) PDLLA/10% gelatin, (**C**) PDLLA/20% gelatin, (**D**) PDLLA/30% gelatin, and (**E**) PDLLA/40% gelatin.

The SEM images of L-929 and MG-63 cells on the PDLLA/gelatin membrane surface after 48 h of culture are shown in Figure 9. Initially, the intermittent attachment of L-929 fibroblasts and MG-63 cells to the hydrophobic PDLLA membrane was observed. As the gelatin content increased, the two cell types began to aggregate, owing to the improved hydrophilicity, and spread extensively across the membrane surface, resulting in superior cell adhesion and proliferation. Although cell adhesion increased, the degree of disintegration increased to 34% due to the hydrophilic nature of the PDLLA/gelatin (40%) membrane. Gelatin hydrogels can retain large amounts of water within their networks without dissolution, forming fine, gelatinous networks [17,18]. Gelatin consists of polypeptide chains bonded together via hydrogen bonds between amino acids in adjacent chains. This semi-rigid material is formed by dispersing a liquid phase through a solid network. The helical junction regions act as bridges, allowing a fine, gelatinous network to form [17,18]. The physically cross-linked bonds of gelatin molecules break down as the temperature increases above the sol–gel transition temperature (35 °C), which is equivalent to the oral state, and the structural integrity of the 3D construct collapses [17,18,19]. The gelatin concentration in PDLLA/gelatin membranes was found to be responsible for their mechanical and physical properties, cell adhesion, and degradability. 

BP PDLLA/gelatin membranes were electrospun using the HFIP solvent to determine the wet mechanical and chemical properties and biocompatibility of the PDLLA/gelatin blend membranes as a function of the gelatin concentration. Regardless of the environment, the stress and strain at the break of the membrane increased and decreased, respectively, with increasing gelatin content. Among the investigated membranes, PDLLA/gelatin hybrid membranes with gelatin content in the range of 10 to 20 wt% are likely to be used as GBR inducers due to their tunable wet mechanical properties (σ_w_ = 5.4–6.3 MPa, ε_w_ = 2.9–3.0), WUC (337–571%), degradability (11.4–20.2%), and excellent biocompatibility. Except for van der Waals interactions, no chemical bonding between the PDLLA and gelatin chains was observed. The gelatin content of PDLLA/gelatin membranes is attributed to their wet mechanical and physical properties, cell viability, adhesion, proliferation, and degradability.

## 3. Materials and Methods

### 3.1. Materials 

PDLLA (Resomer^®^ R207S, St. Louis, MO, USA) and gelatin from porcine skin (gel strength 300 bloom, Type A, St. Louis, MO, USA) were purchased from Sigma-Aldrich, USA. HFIP solvent was purchased from Daejung Chemicals & Metals Co., Ltd. (Cheongju, Chungbuk, Republic of Korea) and used as received without further purification. For formulations containing gelatin, both polymers were dissolved in HFIP over a range of total polymer (PDLLA + gelatin) concentrations from 3 to 9 wt%. PDLLA was mixed with gelatin in the composition range of 0–40 wt%. The PDLLA concentration for electrospinning with HFIP was optimized based on strength, strain at break, and WUC.

### 3.2. Membrane Synthesis and Characterization 

Membranes were synthesized using electrospinning equipment consisting of a syringe pump (KDS-200, Stoelting Co., Wood Dale, IL, USA), BD luer-lock syringe, metal needle, grounded drum collector (NanoNC Co., Ltd., Seoul, Republic of Korea), and a high-voltage power supply (ES30P-5W, Gamma High Voltage Research Inc., Ormond Beach, FL, USA) [7,10,13,14]. The PDLLA/gelatin precursor solution was placed in a 10 mL BD luer-lock syringe attached to a syringe pump (EP100, NanoNC Co., Ltd., Seoul, Republic of Korea) and fed into a 20-gauge metal needle at a flow rate of 1 mL/h. The PDLLA/gelatin fibers were collected using a rotating drum collector with a diameter of 9 cm, length of 20 cm, voltage of 20 kV, and distance of 15 cm. The membranes were synthesized as previously described [7,10,13,14]. PDLLA with varying gelatin concentrations, ranging from 0 to 40 wt%, was prepared by dissolving gelatin in HFIP for 3 h at room temperature (RT) using a magnetic stirrer. 

The viscosity of the gelatin/HFIP solution was measured at 25 °C using a viscometer (DV 1M, Brookfield, Middleboro, MA, USA) with spindle No. SC4-31 at 30 rpm [7,10,13,14,32]. The chemical bonding of the PDLLA/gelatin polymers was studied using Fourier transform infrared spectroscopy (Spectrum Two, PerkinElmer, Beaconsfield, UK). Measurements were taken in absorption mode at 2 cm^−1^ intervals and within the wavelength range of 4000–400 cm^−1^, as previously described [7,10,13,14]. Membrane surface morphology was examined using an SEM (S-3000H, Hitachi, Tokyo, Japan) and an optical stereomicroscope (SV-55, Sometech Inc., Seoul, Republic of Korea). The fiber diameter was assessed using an optical microscope equipped with i-Solution Lite image software (ver. 9.1; IMT *i*-Solution, Inc., Vancouver, BC, Canada), as previously described [7,10,13,14].

The tensile strength of the membranes was tested using an Instron 5564 (Norwood, MA, USA) at a crosshead speed of 10 mm/min. Dumbbell-shaped specimens were prepared according to ASTM D-638 (Type V). All the experiments were performed at least five times. Freeze-dried membranes were cut, weighed, and immersed in 0.01 M phosphate-buffered solution (PBS) containing 0.1 mg/mL of lysozyme (Muramidase from hen egg white, Roche Diagnostic GmbH, Mannheim, Germany). The samples were incubated in a simulated body environment, that is, at 37 °C and 80 rpm. Lyophilized samples were collected every seven days and then completely freeze-dried and weighted to evaluate weight loss due to enzymatic degradation. The in vitro enzymatic degradation rate was determined using the following equation:

(1) $$\mathrm{degradation}\;\mathrm{rate}\;\left(\%\right)=\frac{W_{i}-W_{f}}{W_{i}}\times 100$$

where *W_i_* and *W_f_* are the initial and final weights of the membrane, respectively [7,10]. The membrane WUC was determined as previously described [7]. The WCA was measured using a droplet analyzer (SmarDrop Standard, FEMTOFAB, Seongnam, Gyeonggi-do, Republic of Korea) [8,10]. The experimental results were expressed as mean ± standard deviation, and *p* < 0.05 was considered statistically significant [10,13,14].

### 3.3. Cell viability, Proliferation, and Attachment

The cytotoxic potential of PDLLA/gelatin membranes was assessed using extraction assays performed according to the International Organization for Standardization (ISO 10993-5) guidelines [7,10,29]. L-929 (NCTC Clone 929; ATCC, Manassas, VA, USA) and MG-63 (Korea Cell Line Bank, Seoul, Republic of Korea) cells were used. The extract was aseptically collected from the medium, with a sample-to-surface area to the extracting solvent volume (cm^2^/mL) ratio of 6:1 (test extracts). The experimental procedures were conducted as previously described [7,10,13,14,29,31].

For cell proliferation, MC3T3-E1 (mouse-calvaria-derived preosteoblasts; ATCC, Manassas, VA, USA) and L-929 cells were seeded in a 24-well plate (2 × 10^4^ cells/well) and incubated at 37 °C and 5% CO_2_ for 24 h for cell attachment. Following incubation, the culture medium was replaced with the extract-containing medium and further incubated. At 12, 24, and 48 h, the attached cells were stained using the Live/Dead^TM^ Viability/Cytotoxicity Kit (Thermo Fisher Scientific, Waltham, MA, USA), according to the manufacturer’s instructions. Green represents living cells, and red represents dead cells. Images were obtained using a fluorescence microscope (EVOS FL; Thermo Fisher Scientific, Waltham, MA, USA) [8,10,15].

To determine L-929 and MG-63 cell affinity to the membrane, 1 × 10^4^ cells/well were seeded on PDLLA/gelatin membranes and incubated for 48 h at 37 °C with 5% CO_2_ [7,10]. Cells attached to the membrane surface were fixed using 4% formaldehyde (Daejung Chemicals & Metals, Cheongju, Chungbuk, Republic of Korea) in PBS for 20 min at RT and then with methanol (Samchun Chemicals, Seoul, Republic of Korea) for 5 min at −20 °C [8]. PBS 1X was prepared by mixing 137 mM NaCl, 2.7 mM KCl, 10 mM Na_2_HPO_4_, and 1.8 mM KH_2_PO_4_, purchased from Samchun Chemicals, in distilled water. To assess cell adhesion, PLLA/gelatin membrane morphology was examined using SEM (S-3000H, Hitachi, Tokyo, Japan).

## 4. Conclusions

We investigated the effect of gelatin content on the wet mechanical properties of PDLLA membranes by electrospinning 5 wt% PDLLA/gelatin BP membranes with gelatin content ranging from 0 to 40 wt%. Regardless of the environment, fracture stress increased and strain decreased with increasing gelatin content. Due to the hydrophilic nature of gelatin, wet strains consistently outperform dry strains. Conversely, wet stress is always lower than dry stress due to hydrolysis-induced biodegradation. PDLLA/gelatin membranes can be used as GBR inducers regardless of gelatin content due to their suitable wet mechanical properties (4.5~8.6 MPa) and degradability (1.8~34%). In addition, excellent biocompatibilities were also verified by examining cytotoxicity, cell proliferation, and adhesion. 

PDLLA is a non-piezoelectric polymer, given its chemical configuration. Nowadays, biocompatible and biomimetic materials for bone tissue engineering have emerged, promoting cell and tissue growth in vitro and in vivo [37,38]. Piezoelectric poly(L-lactic acid) (PLLA) BPs can mimic the piezoelectric properties of bone, helping bone repair by converting physiological mechanical signals into electrical signals and promoting electrical potential on the surface of bone tissue. Piezoelectricity can enhance cell adhesion, proliferation, and osteogenic differentiation of bone marrow mesenchymal stem cells. Therefore, periodontal barriers based on piezoelectric materials, combined with bone grafts, may effectively enhance healing in fibrous connective tissues, bone tissues, and marrow tissues. However, further in vivo studies on piezoelectric biodegradable barrier membranes, combined with bone grafts [34] containing growth factors [39], antibiotics [36,40], and regenerative cells [41], are needed for a comprehensive clinical evaluation.

## Data Availability

The data presented in this study are available on request from the corresponding author.

## Supplementary Materials

The following supporting information can be downloaded at: https://www.mdpi.com/article/10.3390/ijms25095022/s1.
