# Supplementary material for: Gelatin Enhances the Wet Mechanical Properties of Poly(D,L-Lactic Acid) Membranes
Source: Int J Mol Sci. 2024 May 4;25(9):5022. doi: 10.3390/ijms25095022 (PMC11084932; doi:10.3390/ijms25095022)
Supplement: Supplementary file 1 [file ijms-25-05022-s001.zip › ijms-2968130-supplementary.pdf]

Support Information of: Gelatin Enhances the Wet Mechanical Properties of Poly(D,L-Lactic Acid) Membranes

Deuk Yong Lee [1,2,*]

[1] Department of Biomedical Engineering, Daelim University, Anyang 13916, Korea; duke1208@gmail.com (D.Y.L.)
[2] Department of R&D Center, Hass Co., Ltd., Gwangmyeong 14322, Korea; duke1208@gmail.com (D.Y.L.)
* Correspondence: duke1208@gmail.com (D.Y.L.)

Supporting Figures:

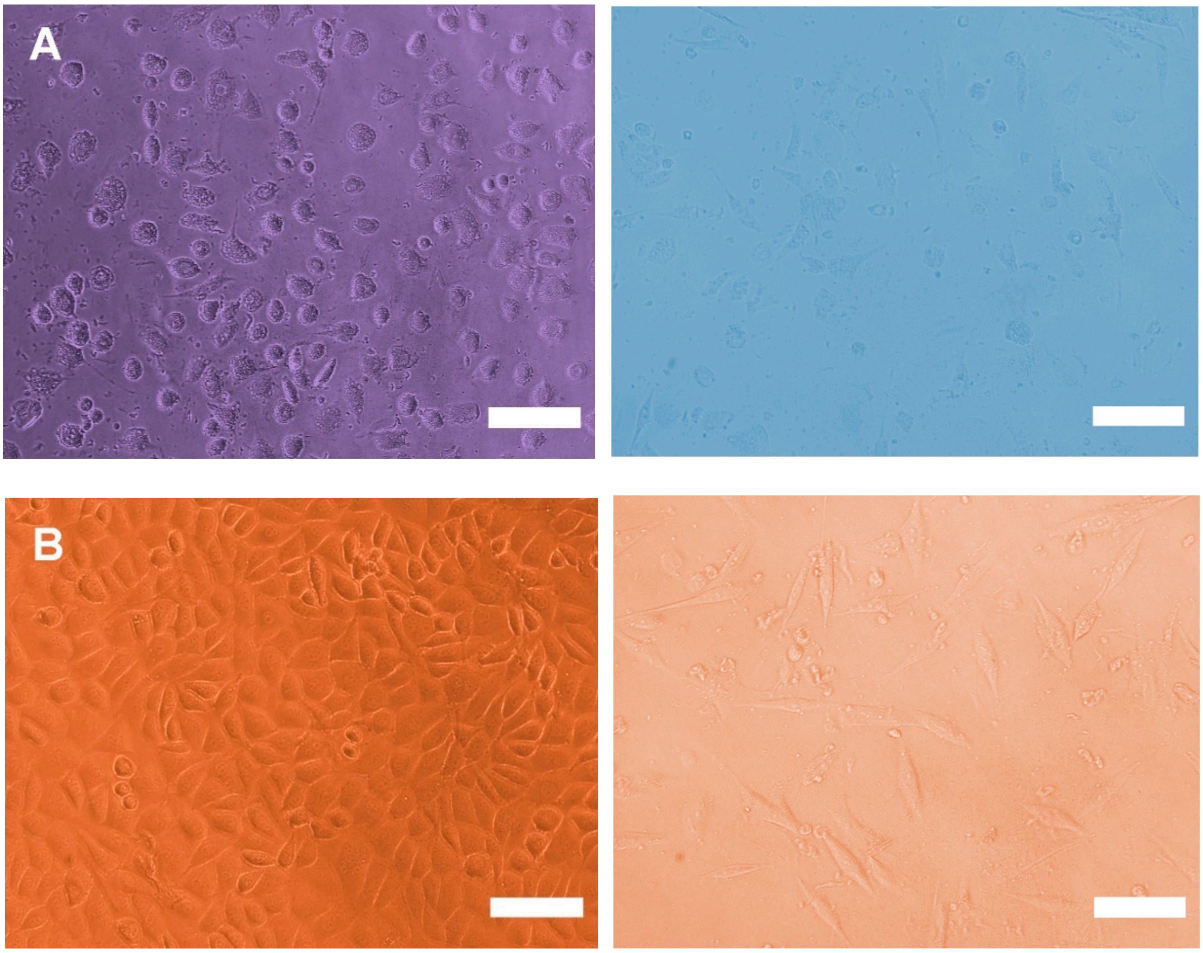

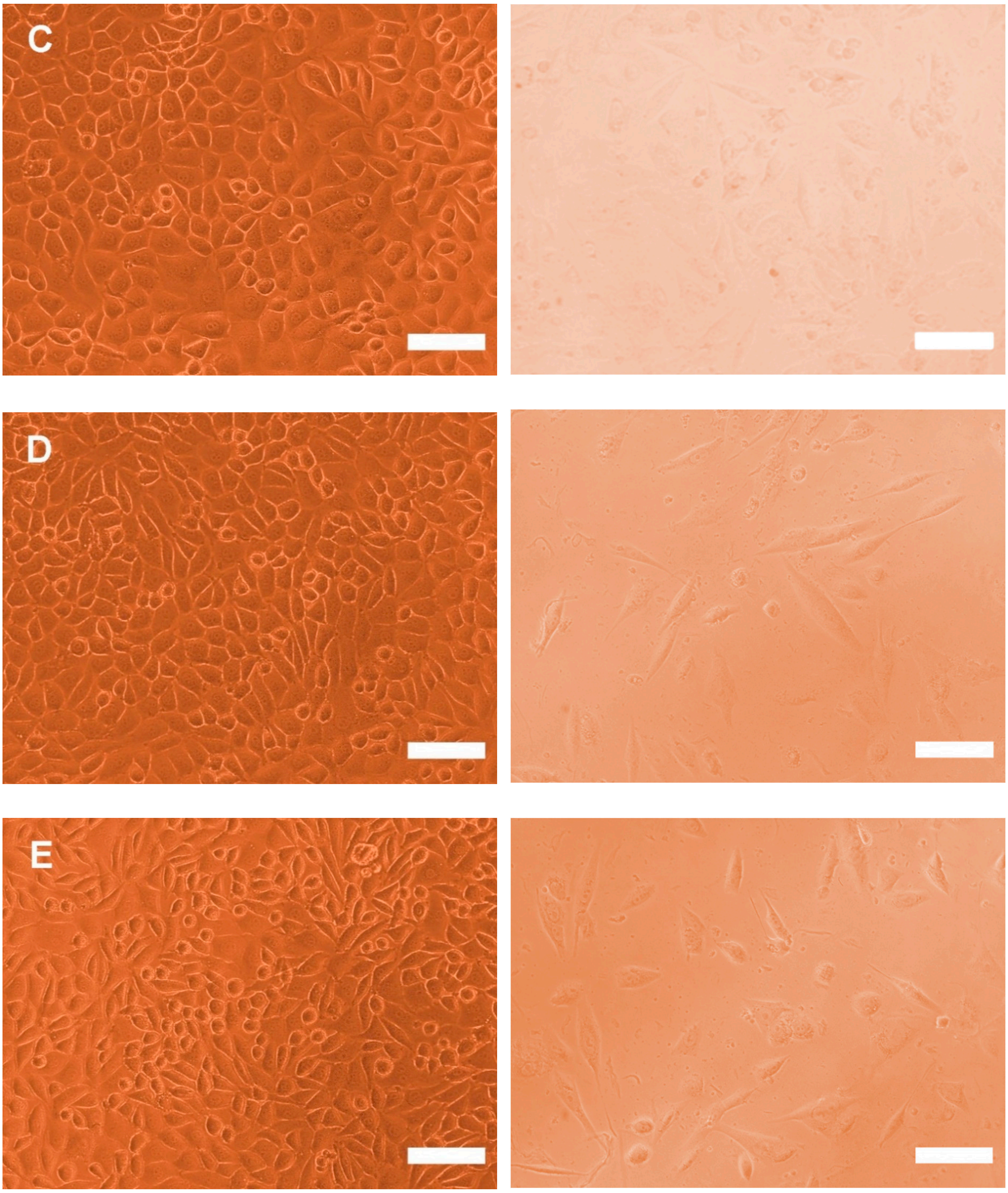
C
D
E

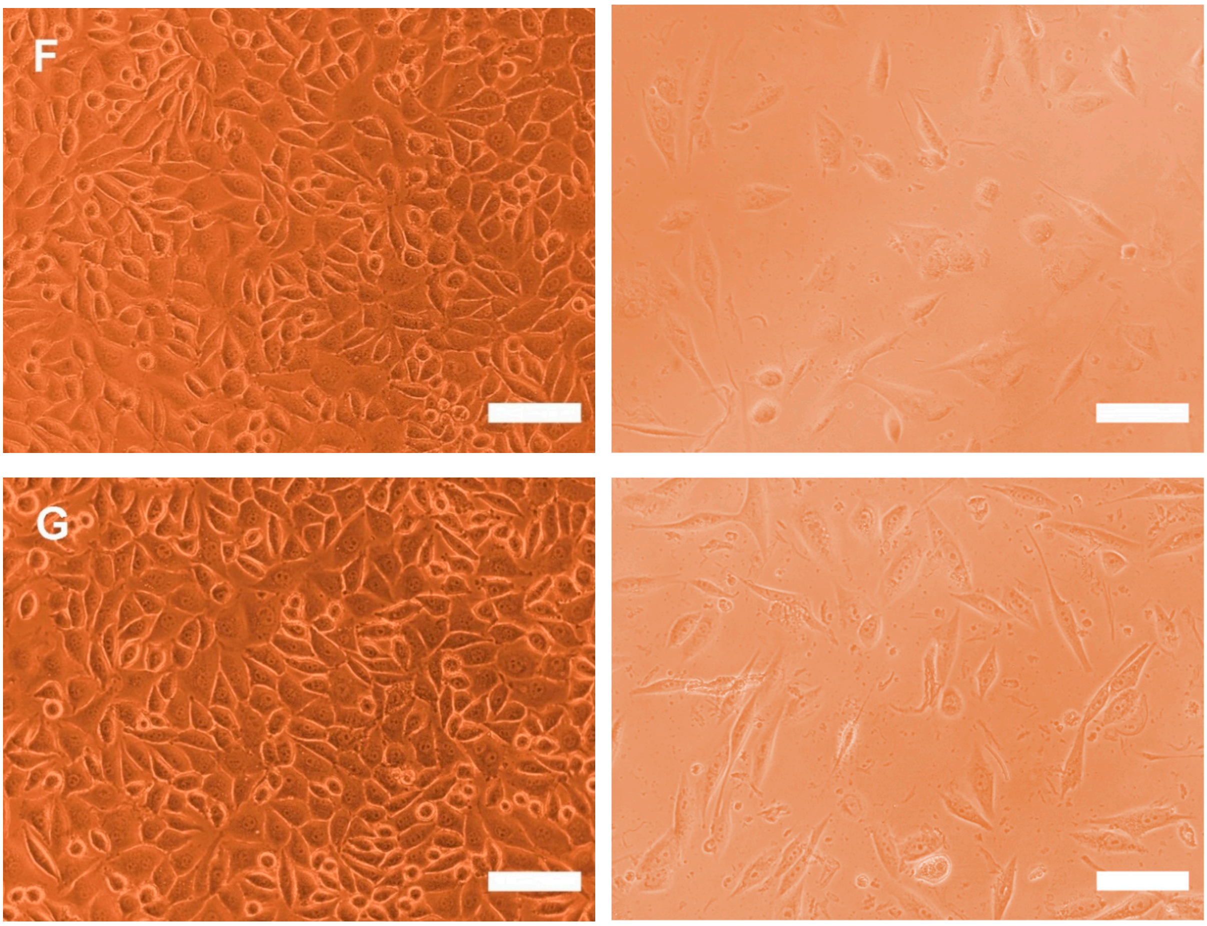

Figure S1. Photographs of L-929 and MG-63 cell morphology: **A** positive control, **B** negative control, and the extracts of poly(D,L-lactic acid)/gelatin membranes as a function of gelatin concentration: **C** 0%, **D** 10%, **E** 20%, **F** 30%, and **G** 40% from EZ-cytox after exposure to the membrane suspensions for 48 h. Scale bar = 100 μm.

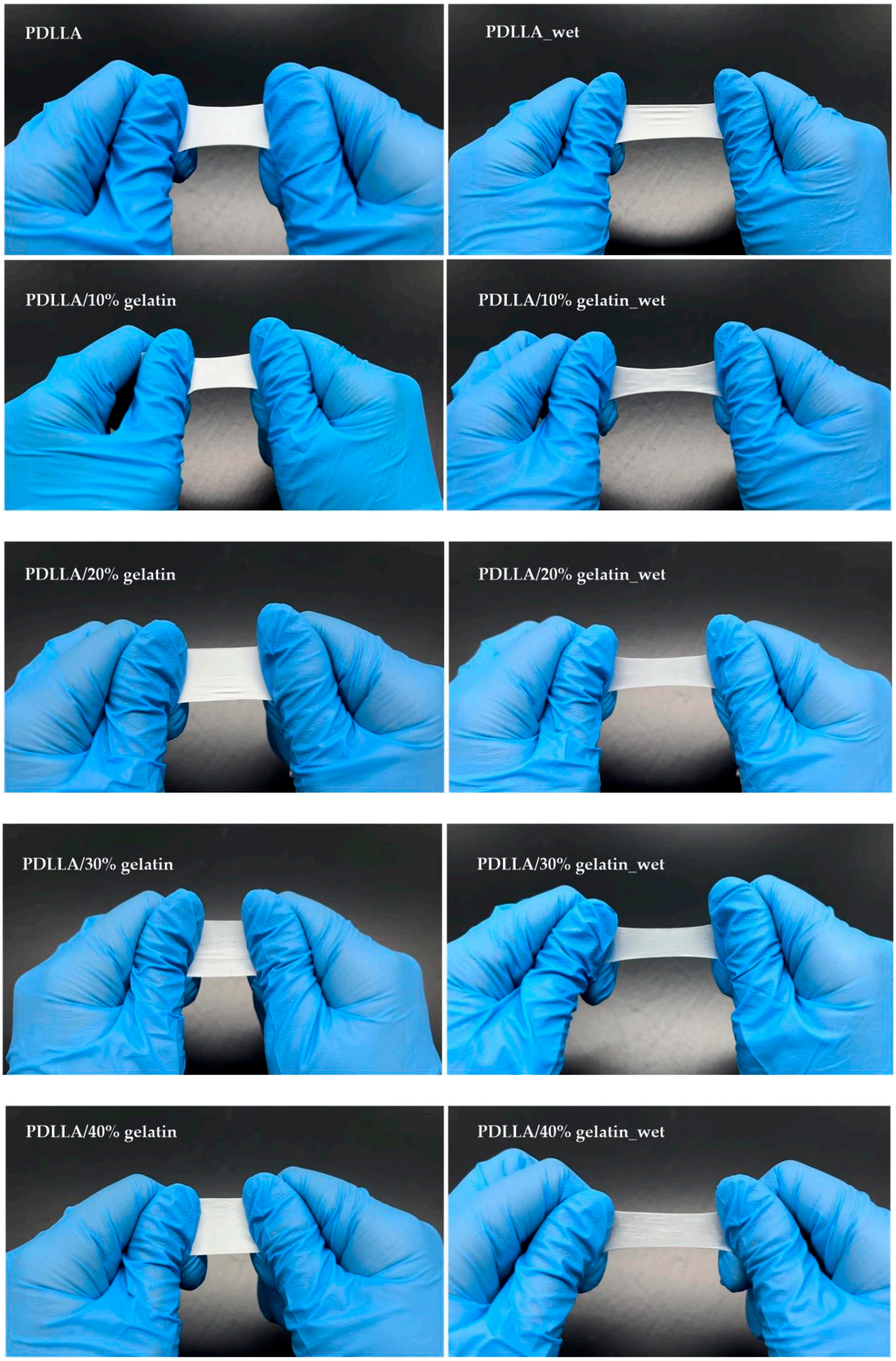

Figure S2. Finger-stretched optical photographs of various poly(D,L-lactic acid)/gelatin membranes in dry and wet conditions.

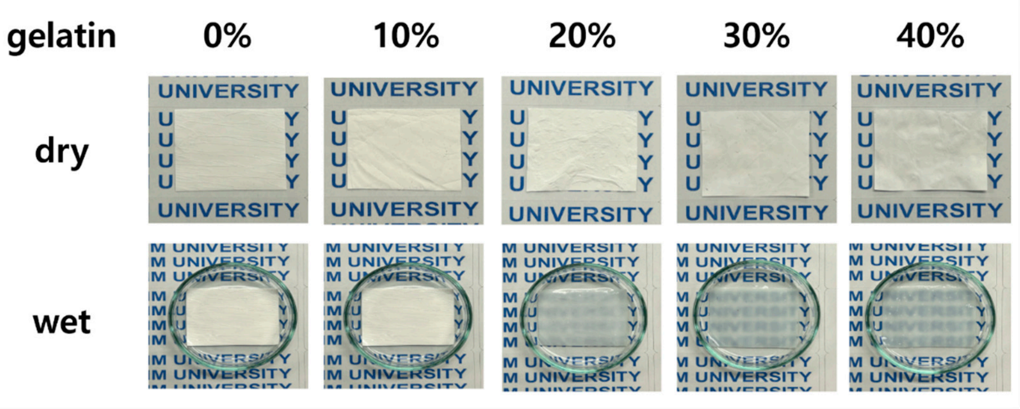

Figure S3. Photographs of poly(D,L-lactic acid)/gelatin membranes with varying gelatin contents in dry and wet states.
